# Supplementary material for: The 3′UTR VNTR SLC6A3 Genetic Variant and Major Depressive Disorder: A Systematic Review
Source: Biomedicines. 2023 Aug 15;11(8):2270. doi: 10.3390/biomedicines11082270 (PMC10452352; doi:10.3390/biomedicines11082270)
Supplement: Supplementary file 1 [file biomedicines-11-02270-s001.zip › biomedicines-2489234-supplementary.pdf]

*Systematic Review*

# The *3′UTR VNTR SLC6A3* genetic variant and Major Depressive Disorder: A systematic review

**Bruna Rodrigues Gontijo[1], Isabella Possatti[1], Caroline Ferreira Fratelli[1], Alexandre Sampaio Rodrigues Pereira[1], Larissa Sousa Silva Bonasser[2], Calliandra Maria de Souza Silva[1] and Izabel Cristina Rodrigues da Silva[1,*]**

[1] Graduate Program in Health Sciences and Technologies, Faculty of Ceilandia, University of Brasilia, Federal District, Brasilia 72220-900, Brazil; brunargontijo.unb@gmail.com (B.R.G.); isabellapossatti@gmail.com (I.P.); carolfratelli@gmail.com (C.F.F.); prof.alexandresampaio@gmail.com (A.S.R.P.); cdssilva@gmail.com (C.M.d.S.S.)

[2] Graduate Program in Health Sciences, University of Brasilia, Federal District, Brasilia 70910-900, Brazil; laribonasser@gmail.com

* Correspondence: belbiomedica@gmail.com; Tel.: +55-(61)-3107-8400

**Supplementary Material**

**Table S1.** The systematic review selected articles' quality assessment according to the Genetic Risk Prediction Studies (GRIPS) guideline.

**Table S1.** The systematic review selected articles' quality assessment according to the Genetic Risk Prediction Studies (GRIPS) guideline.

| **Section/Topic** | | Frisch et al. [18] | Kirchheine, J., et al. **[19]** | Lavretsky et al. [20] | Huang, Chang-Chih et al. [21] | Rafikova E. I. et al. [22] | Rafikova, E.I., et al. [14] |
|---|---|---|---|---|---|---|---|
| **Methods** | | | | | | | |
| **Study desing and Setting** | 4) Present key elements of study design early in the paper and Describe the setting, locations, and relevant dates, including periods of recruitment, exposure, follow-up, and data collection | | x | x | x | x | x |
| **Participants** | 5) Describe eligibility criteria for participants, and sources and methods of selection of participants | x | x | x | x | x | x |
| **Variables: Definition** | 6) Clearly define all participant characteristics, risk factors and outcomes. Clearly define genetic variants using a widely-used nomenclature system | x | x | x | x | x | x |
| **Variables: Assessment** | 7) (a) Describe sources of data and details of methods of assessment (measurement) for each variable. | | x | x | | x | x |
| | (b) Give a detailed description of genotyping and other laboratory methods. | x | x | x | x | x | x |
| **Variables: Coding** | 8) (a) Describe how genetic variants were handled in the analyses | x | x | | x | x | x |
| | (b) Explain how other quantitative variables were handled in the analyses. If applicable, describe which groupings were chosen, and why. | x | x | x | x | x | x |

| | | | | | | | |
|---|---|---|---|---|---|---|---|
| **Analysis: Risk model construction** | 9) Specify the procedure and data used for the derivation of the risk model. Specify which candidate variables were initially examined or considered for inclusion in models. Include details of any variable selection procedures and other model-building issues. Specify the horizon of risk prediction (e.g., 5-year risk). | x | x | x | x | x | X |
| **Analysis: Validation** | 10) Specify the procedure and data used for the validation of the risk model. | | x | x | x | x | x |
| **Analysis: Missing data** | 11) Specify how missing data were handled | | x | | | | |
| **Analysis: Statistical methods** | 12) Specify all measures used for the evaluation of the risk model including, but not limited to, measures of model fit and predictive ability | | x | x | x | x | x |
| **Analysis: Other** | 13) Describe all subgroups, interactions, and exploratory analyses that were examined | x | x | x | x | x | x |

X - present

**Table S1.** *Cont.*

| Section/Topic | | Frisch et al. [18] | Kirchheine, J., et al. **[19]** | Lavretsky et al. **[20]** | Huang, Chang-Chih et al. **[21]** | Rafikova E. I. et al. **[22]** | Rafikova, E.I., et al. **[14]** |
|---|---|---|---|---|---|---|---|
| **Results** | | | | | | | |
| **Participants** | 14) Report the numbers of individuals at each stage of the study. Give reasons for nonparticipation at each stage. Report the number of participants not genotyped, and reasons why they were not genotyped. | | x | x | | x | x |
| **Descriptives: Population** | 15) Report demographic and clinical characteristics of the study population, including risk factors used in the risk modeling. | x | x | x | x | x | |
| **Variables: Definition** | 16) Report unadjusted associations between the variables in the risk model(s) and the outcome. Report adjusted estimates and their precision from the full risk model(s) for each variable. | x | x | x | x | x | x |
| **Descriptives: Model estimates** | 17) Descriptives: Model estimates | x | x | x | x | x | x |
| **Assessment** | 18) Report measures of model fit and predictive ability, and any other performance measures, if pertinent. | | x | x | x | x | x |
| **Validation** | 19) Report any validation of the risk model(s) | x | x | x | x | x | X |
| **Other analyses** | 20) Present results of any subgroup, interaction, or exploratory analyses, whenever pertinent | x | x | x | x | x | X |

| | | | | | | | |
|---|---|---|---|---|---|---|---|
| **Discussion** | | | | | | | |
| **Limitations** | 21) Discuss limitations and assumptions of the study, particularly those concerning study design, selection of participants, and measurements and analyses, and discuss their impact on the results of the study | x | x | x | x | x | x |
| **Interpretation** | 22) Give an overall interpretation of results considering objectives, limitations, multiplicity of analyses, results from similar studies, and other relevant evidence | x | x | x | x | X | x |
| **Generalizability** | 23) Discuss the generalizability and, if pertinent, the health care relevance of the study results | x | x | | x | x | x |

X - present
